# Relentless Reflection

**DOI:** 10.1055/s-0045-1809700

**Published:** 2025-07-01

**Authors:** S. Raja Sabapathy

**Affiliations:** 1 Department of Plastic Surgery, Hand and Reconstructive Microsurgery and Burns, Ganga Hospital, Coimbatore, Tamil Nadu, India

The invitation to write the editorial for our journal had some thought-provoking statements and requisitions. It spoke of “the responsibility of mentoring the next generation and guiding them toward achieving meaningful success and obtaining global recognition.” It requested me to *reflect* , inspire, and chart the path ahead for the future of plastic surgery. It read, “your *reflections* on clinical practice, research, and the practical considerations of building a successful plastic surgery practice will serve as a guiding light for young minds.” I understood that I had a tough task on hand.

Coincidentally, about a fortnight ago, I had the privilege of having dinner with an icon of the Indian automobile industry. He has led companies which are a pride of our country, which have won many prestigious international awards for excellence in manufacturing and customer satisfaction. When discussing the path to excellence in manufacturing, he repeatedly used the phrase “ *Relentless Reflection* .” He said that, accepting that there is room for improvement is an essential trait to have for progress to occur. He advised that we must benchmark our work against the world's best, focus on the customer's expectations, and implement change wherever needed. He extolled us to *relentlessly reflect* every day, and in every one of our actions, to see if they meet the world standards.

This inspired me to title this piece “Relentless Reflection.” I have attempted to reflect on the past, assess the present, and guide to prepare for the future. We are in a specialty, where our actions could make a great difference in the lives of people. We deal with ailments ranging from trauma to cancer, burns to congenital anomalies. There is not a domain in the human body that we don't touch. The need for our services in the society is huge. Still, most plastic surgeons feel that they should get to do more. There is a fear of losing out to sister specialities. This fear drives many to despair. I believe that somewhere along the way, we missed connecting the dots.

There were three points that the automobile industry leader touched upon that night which really struck me. First, if you desire a change to happen, it has to start with you. Second, in every aspect of your work you need to aim for the best. Third, excellence is a continuous process.

It is time that each one of us trains our mind for “relentless reflection,” to check if our work matches with what is the best possible. Thinking back, when we started in 1991, at Ganga Hospital, I realize that instinctively we did work that way. Even when we were small, every time we received a new patient, I would constantly think about ways to achieve as good an outcome as was achieved at Louisville, where I was trained. We benchmarked our work against the best centre, at that time. We did not have computed tomography (CT) scans, intensive care units, or even a blood bank. Yet, when we had patients with hand amputation and concomitant head injury, we found ways to get CT scans, enlist the services of a neurosurgeon, and still make the replant possible! In fact, we had a series of patients with extradural hemorrhage who also underwent microsurgery for hand trauma. We must continuously reflect upon how we overcome the odds and do our best. Undoubtedly, the constraints are real. But overcoming those constraints makes us champions. And our specialty needs champions. Each of us must aim to be one.

Another aspect of bettering our field is attracting top talent to plastic surgery. Frequently, we hear that our specialty is undersold in terms of its image. Hence, undergraduates and postgraduates remain “plastic surgery dark.” It makes me think, what we can do to change this. Our young doctors in medical schools get inspired by what they see happening in the hospitals they are being trained at. That is more compelling than the tons of images one can put on the social media. General surgery postgraduates rotate through the super specialities and that offers them an opportunity to form impressions first hand of the scope and future of the specialty. If we aim to make every plastic surgery unit in teaching hospitals, the most respected super specialty unit of the institution, we will surely attract the brightest young minds into the specialty. Decades ago, we were disadvantaged in not having technologically advanced equipment used on a regular basis. Now we have them all. Nothing could be more striking to see than a well-functioning hand transplant or a major replant or a badly burnt face reconstructed or mutilated hands made functional. We must remember that not all decisions run, based on economics. Once quality is ensured, everything, even economics will fall in place.

Now, I would like to reflect on the need of expanding the realm of plastic surgery versus the fear of losing out some of our territories of practice. This is a worldwide concern and not just an Indian problem. Years ago, Peter Neligan and I shared the stage at a symposium in Vancouver, on the prospect of plastic surgeons losing out to other specialities. Peter spoke very well, with examples from other fields. The main thrust of his talk was on the value of embracing change. When interventional cardiology evolved, it was thought that cardiac surgeons would naturally take it up. But that did not happen. Many cardiac surgeons thought it below their standards to perform interventional procedures and felt that as surgeons they should only perform surgeries. They did not see the future coming. Cardiac surgeons had a window of opportunity to take up interventional work when the cardiologists had not even started. The result of that is out there for everyone to see. Peter then said that specialities lose out when we overlook the patient's expectations and their needs, and we just choose to be in our comfort zones. He went on to give examples to show that keeping up with advances, innovating, and catering to what really the patients need, is the only way for specialties to survive and grow. Reflecting upon this, he went on to say, that it is the responsibility of the leaders in the field to embrace change, and to take up new subspecialties as they evolve. An important milestone to this effect was us taking up microsurgery, as it came along. Perhaps, we should have taken up aesthetic surgery as part of the routine work in all the teaching institutions a decade ago. That was a very thought-provoking speech, in my opinion.

When I was being trained in the 1980s, cleft lip and palate were only repaired by the plastic surgeons. During my training days at a conference dinner, some professors were informally discussing about the cleft load in their units. One said that they had a waiting list for over a year, and another said that he no longer looks at his register, since it has become so voluminous. Another added that their unit didn't even have time for providing surgery, let alone thinking about speech therapy. I think that was a time when we failed to pick up on a leadership opportunity. If only we had worked hard then, collaborated to establish institutions which offered comprehensive cleft care, we would have remained the leaders in the field. *We did what we could but not what was needed* . The need-gap was not addressed by us as a community. This became apparent when some plastic surgeons operated hundreds of cleft children every month when Smile Train was introduced. History will keep on repeating itself, in all the fields in the same way be it burn care, onco-reconstruction, diabetic foot surgery, or management of lymphedema.

Due to our extensive training, we are capable and are poised for greatness. But if we don't take efforts to gain fresh skills, meet patient expectations, and provide comprehensive care, we cannot hold ground. Hand surgery is an example. Plastic surgeons equated “hand injury” to “hand surgery.” We were good in soft tissue cover and acute trauma but that is only a small facet of hand surgery. If one peruses Green's Operative Hand Surgery textbook, the chapters related to hand injury and reconstruction occupy only 25% of the book. There is another 75% which is “hand surgery” and which we did not choose to take care of. The demands of a present-day hand surgeon, say, when he treats a manual worker with dorsal composite tissue loss or a professional cricketer who has a comminuted intra-articular fracture of the proximal interphalangeal joint are different. Advancing to deliver that care and to be at the cutting edge of the care pathway in sports injuries, spastic hands, wrist problems, or complex congenital differences needs extra training. Only very few plastic surgeons venture into specializing and that is a cause for concern. With advances taking place at a rapid pace, the requirement to take up postdoctoral fellowships is mandatory for us to retain leadership across all our subspecialities. It is impossible to contain knowledge. The pressure to fill up the need-gap will overcome territorial ambitions and specialty boundaries, if we don't take up the responsibility to excel and fill it.

Nowadays, with travel becoming easier, access to quality overseas and inland fellowships have opened fresh avenues for training opportunities. There are ever so many educational and technique training videos available for free. Up-skilling is easier today, and it must be done. When access to knowledge is free, what will help us stand out is delivering what our patients need, at the time they need it.

Thinking about gaining global recognition for our work, I believe the way is to contribute to science. When I was the Editor in Chief of the *Journal of Hand Surgery* (Asia Pacific), I was pleased to include an invited article from Jin Bo Tang on what ails hand surgery in the Asia-Pacific region. One of the things he said was that, though we have the numbers we do not reflect on our results. He used the phrase “to inject science into what we do” and emphasized that it was the need of the hour. Reflecting upon our outcomes, making the change needed to bring out the best outcomes, and communicating that to the world will take us to the world stage and not just the absolute numbers.

On November 15, 2018, in Seattle, Jeff Bezos the founder of Amazon in his address to his employees, stunned them when he said, “One day Amazon will fail. Amazon is not too big to fail, but our job is to delay it as long as possible.” The key to prolonging the demise Jeff Bezos continued, “is to obsess over customers and avoid looking inward worrying over itself. If we start to focus on ourselves instead of focusing over our customers that will be the beginning of the end.” I think this applies to all fields. If we become obsessed with patient care, ourselves, our units, and our specialty will automatically grow.

I will complete this piece with two quotes—one from Swami Vivekananda. He said, “Things do not grow better. They remain as they are. It is we who grow better by the changes we make in ourselves.” That goes for up-skilling. The other quote is the advice I got from Harold Kleinert when I left Louisville. He said, “If you *love* hand surgery and keep on doing *good* hand surgery everything will take care of itself.” That goes for patient care. If we relentlessly reflect on what we do every day and analyze if we meet world standards, then we will ever remain at the top.

